# The global burden of melanoma: results from the Global Burden of Disease Study 2015

**DOI:** 10.1111/bjd.15510

**Published:** 2017-06-12

**Authors:** C. Karimkhani, A.C. Green, T. Nijsten, M.A. Weinstock, R.P. Dellavalle, M. Naghavi, C. Fitzmaurice

**Affiliations:** ^1^ Department of Dermatology University of Colorado Aurora CO U.S.A; ^2^ QIMR Berghofer Medical Research Institute Brisbane Queensland Australia; ^3^ CRUK Manchester Institute University of Manchester Manchester U.K; ^4^ Erasmus MC Cancer Institute Department of Dermatology Erasmus University Medical Center Rotterdam Rotterdam the Netherlands; ^5^ Center for Dermatoepidemiology Department of Veterans Affairs Medical Center Providence RI U.S.A; ^6^ Department of Dermatology and Epidemiology Brown University Providence RI U.S.A; ^7^ Dermatology of Epidemiology Colorado School of Public Health Aurora CO U.S.A; ^8^ Dermatology Service U.S. Department of Veterans Affairs Eastern Colorado Health System Denver CO U.S.A; ^9^ Institute for Health Metrics and Evaluation Seattle WA U.S.A; ^10^ Department of Medicine Division of Hematology University of Washington Seattle WA U.S.A

## Abstract

**Background:**

Despite recent improvements in prevention, diagnosis and treatment, vast differences in melanoma burden still exist between populations. Comparative data can highlight these differences and lead to focused efforts to reduce the burden of melanoma.

**Objectives:**

To assess global, regional and national melanoma incidence, mortality and disability‐adjusted life year (DALY) estimates from the Global Burden of Disease Study 2015.

**Methods:**

Vital registration system and cancer registry data were used for melanoma mortality modelling. Incidence and prevalence were estimated using separately modelled mortality‐to‐incidence ratios. Total prevalence was divided into four disease phases and multiplied by disability weights to generate years lived with disability (YLDs). Deaths in each age group were multiplied by the reference life expectancy to generate years of life lost (YLLs). YLDs and YLLs were added to estimate DALYs.

**Results:**

The five world regions with the greatest melanoma incidence, DALY and mortality rates were Australasia, North America, Eastern Europe, Western Europe and Central Europe. With the exception of regions in sub‐Saharan Africa, DALY and mortality rates were greater in men than in women. DALY rate by age was highest in those aged 75–79 years, 70–74 years and ≥ 80 years.

**Conclusions:**

The greatest burden from melanoma falls on Australasian, North American, European, elderly and male populations, which is consistent with previous investigations. These substantial disparities in melanoma burden worldwide highlight the need for aggressive prevention efforts. The Global Burden of Disease Study results can help shape melanoma research and public policy.

## Abstract 2

**What's already known about this topic?**

- Melanoma incidence and mortality has been assessed in the past for individual countries or world regions.

**What does this study add?**

- As part of the Global Burden of Disease Study, melanoma burden was estimated at the global, regional and country level for incidence, mortality, prevalence, years lived with disability, years of life lost and disability‐adjusted life years.
- These estimates can be used to guide prevention and treatment strategies, as well as resource allocation.

Respond to this article

Plain language summary available online

The landscape of melanoma, the most deadly skin cancer, has changed dramatically in the twenty‐first century. Prevention, including increased public education and awareness, early detection, genetic testing and substantial improvements in advanced melanoma therapies are examples of recent progress. To describe fully the effect of a disease on a population, metrics beyond incidence and mortality are needed.^1^ One approach is to estimate disability‐adjusted life years (DALYs), which combine morbidity and mortality metrics.^2^ For reference, one DALY is equivalent to 1 year of healthy life lost. Prior studies have applied DALYs to study melanoma burden among other cancers in world regions and also within individual countries.^3^, ^4^ However, an up‐to‐date large‐scale effort to quantify the comprehensive burden of melanoma, as well as the diversity of causes on global and national scales, is warranted. DALYs, in addition to standard metrics such as incidence, mortality and prevalence are estimated as part of the Global Burden of Disease Study (GBD). The GBD is a systematic scientific effort to quantify the comparative magnitude of health loss resulting from diseases, injuries and risk factors according to age, sex and geography over time.^5^, ^6^ For GBD 2015, the burden of 310 diseases, including melanoma, was estimated.^7^ This study presents the GBD 2015 melanoma incidence, mortality and DALY estimates by sex for 21 world regions encompassing 195 countries and territories.

## Materials and methods

Detailed GBD methodology is published elsewhere.^2^, ^8^, ^9^ A brief overview of the specific melanoma estimation strategy is presented here. Data sources include vital registration systems and cancer registry incidence data that were first transformed to mortality estimates using separately estimated mortality‐to‐incidence (MI) ratios. Briefly, incidence and mortality data were matched by cancer, age, sex, year and location. Multiple logit random effects models were tested, comparing mean MI predictions and mean root‐mean‐squared error to determine a final model output. After removing outliers (data points that unrealistically influenced the model) and space‐time smoothing (spatiotemporal regression to smooth residuals over space, time and age), a Gaussian process regression was performed, which interpolates nonlinear trends. Final MI ratios with 95% confidence intervals (CIs) were generated by back‐transforming 1000 draws from the posterior distribution.^8^ Cancer registry data were obtained either by contacting cancer registries directly or accessing publicly available data sources such as CI5 (Cancer Incidence in Five Countries).^10^ Data sources were used as input into a cause‐of‐death modelling tool (i.e. cause‐of‐death ensemble model approach), which combines many possible models into an ensemble with more accurate trends and smaller error than a single model.^6^, ^11^ To improve estimates for melanoma mortality in areas with sparse data, the following covariates were used: income per capita, years of education per capita, latitude, smoking, alcohol intake, animal fat consumption, fruit and vegetable consumption, mean body mass index and diabetes prevalence. Melanoma mortality together with all other single‐cause estimates were adjusted to fit into the separately estimated all‐cause mortality. For melanoma incidence estimates, final melanoma mortality estimates were divided by the MI ratios.

The 10‐year prevalence was estimated using melanoma incidence and survival estimates. Melanoma survival was estimated by transforming MI ratios into an access‐to‐care variable and scaling each incidence cohort between a ‘best case’ and ‘worst case’ survival.^8^ The ‘best case’ survival curve was derived from Surveillance, Epidemiology, and End Results (SEER) programme 2010 data, while the ‘worst case’ survival curve was derived from the 1950 U.S. Mortality Files and Cancer Survival in Africa, Asia, the Caribbean and Central America (SurvCan). Prevalence was divided into the following four disease phases: (i) diagnosis and treatment, (ii) remission, (iii) metastatic and (iv) terminal. A constant duration for the diagnosis and treatment phase (2 months),^12^ metastatic phase (7·18 months)^13^ and terminal phase (1 month) was used for all ages, countries and times, owing to a lack of data regarding stage distribution and treatment in a majority of countries. Prevalence of remission was estimated by subtracting the sum of the remaining phases from the total prevalence by location, age, sex and year. Prevalence estimates were multiplied by distinct disability weights, derived from population surveys and an open access web‐based survey, to generate years lived with disability (YLDs).^14^ Years of life lost (YLLs) were calculated by multiplying the number of deaths in each age group by the corresponding standard life expectancy. The normative standard life expectancy was based on the lowest age‐specific mortality rate from any cause in each year from all countries with populations greater than 2 million people. The normative standard life expectancy for GBD 2015 was 86·59 years.^15^ YLD and YLL estimates were added for each age group, location, sex and year to yield DALYs. All estimates were produced with 95% uncertainty intervals. Melanoma incidence, DALYs and mortality are reported for 195 countries represented by 21 world regions, both sexes and 14 age groups, ranging from 15 to ≥ 80 years (see Table 1 for GBD regions and represented countries).

**Table 1:** Global Burden of Disease Study region classifications

Global Burden of Disease Study region	Countries represented
East Asia	China, North Korea, Taiwan
Oceania	American Samoa, Federated States of Micronesia, Fiji, Guam, Marshall Islands, Northern Mariana Islands, Papua New Guinea, Samoa, Solomon Islands, Tonga, Vanuatu
Southeast Asia	Cambodia, Indonesia, Laos, Malaysia, Maldives, Mauritius, Myanmar, Philippines, Sri Lanka, Seychelles, Thailand, Timor‐Leste, Vietnam
South Asia	Bangladesh, Bhutan, India, Nepal, Pakistan
Central Asia	Armenia, Azerbaijan, Georgia, Kazakhstan, Kyrgyzstan, Mongolia, Tajikistan, Turkmenistan, Uzbekistan
Central Europe	Albania, Bosnia and Herzegovina, Bulgaria, Croatia, Czech Republic, Hungary, Macedonia, Poland, Romania, Serbia, Slovakia, Slovenia
Eastern Europe	Belarus, Estonia, Latvia, Lithuania, Moldova, Russia, Ukraine
North Africa and Middle East	Afghanistan, Algeria, Bahrain, Egypt, Iran, Iraq, Jordan, Kuwait, Lebanon, Libya, Morocco, Palestine, Oman, Qatar, Saudi Arabia, Sudan, Syria, Tunisia, Turkey, United Arab Emirates, Yemen
Western Sub‐Saharan Africa	Benin, Burkina Faso, Cameroon, Cape Verde, Chad, Cote d'Ivoire, The Gambia, Ghana, Guinea, Guinea‐Bissau, Liberia, Mali, Mauritania, Niger, Nigeria, Sao Tome and Principe, Senegal, Sierra Leone, Togo
Southern Sub‐Saharan Africa	Botswana, Lesotho, Namibia, South Africa, Swaziland, Zimbabwe
Eastern Sub‐Saharan Africa	Burundi, Comoros, Djibouti, Eritrea, Ethiopia, Kenya, Madagascar, Malawi, Mozambique, Rwanda, Somalia, South Sudan, Tanzania, Uganda, Zambia
Central Sub‐Saharan Africa	Angola, Central African Republic, Congo, Democratic Republic of the Congo, Equatorial Guinea, Gabon
Tropical Latin America	Brazil, Paraguay
Andean Latin America	Bolivia, Ecuador, Peru
Central Latin America	Colombia, Costa Rica, El Salvador, Guatemala, Honduras, Mexico, Nicaragua, Panama, Venezuela
Caribbean	Antigua and Barbuda, The Bahamas, Barbados, Belize, Bermuda, Cuba, Dominica, Dominican Republic, Grenada, Guyana, Haiti, Jamaica, Puerto Rico, Saint Lucia, Saint Vincent and the Grenadine, Suriname, Trinidad and Tobago, Virgin Islands
Western Europe	Andorra, Austria, Belgium, Cyprus, Denmark, Finland, France, Germany, Greece, Iceland, Ireland, Israel, Italy, Luxembourg, Malta, Netherlands, Norway, Portugal, Spain, Sweden, Switzerland, U.K.
Southern Latin America	Argentina, Chile, Uruguay
North America	Canada, U.S.A.
Asia Pacific	Brunei, Japan, Singapore, South Korea
Australasia	Australia, New Zealand

## Results

The global incidence of melanoma in 2015 was 351 880 cases (95% CI 281 633–445 036) with an age‐standardized rate of five cases per 100 000 persons (95% CI 4–7). Melanoma was responsible for 1 596 262 global DALYs (95% CI 1 293 447–1 982 679) with an age‐standardized rate of 23 DALYs per 100 000 persons (95% CI 18–28). Melanoma was also responsible for 59 782 global deaths (95% CI 47 602–72 671) with an age‐standardized rate of one death per 100 000 persons (95% CI 0·7–1). With 100% representing total burden from all conditions studied by GBD 2015, melanoma was responsible for 0·065% of all DALYs.

The five world regions with the greatest incidence rates were Australasia [54 (95% CI 41–78)], North America [21 (95% CI 16–31)], Western Europe [16 (95% CI 11–20)], Central Europe [8 (95% CI 7–11)] and Eastern Europe [8 (95% CI 6–10)] (Table 2). Similarly, these were the five world regions with the greatest DALY rates: Australasia [152 (95% CI 112–221)], North America [66 (95% CI 51–100)], Eastern Europe [65 (95% CI 51–85)], Western Europe [58 (95% CI 41–76)] and Central Europe [58 (95% CI 44–73)]. Mortality rates were also highest in these five regions: Australasia [6 (95% CI 4–8)], North America [2 (95% CI 2–3)], Eastern Europe [2 (95% CI 2–3)], Central Europe [2 (95% CI 2–3)] and Western Europe [2 (95% CI 1–3)].

**Table 2:** Age‐standardized incidence, disability‐adjusted life year (DALY) and mortality rates for 21 world regions

Region	Incidence rate (95% CI)	DALY rate (95% CI)	Mortality rate (95% CI)
Australasia	54·1170 (40·7429–77·8116)	151·5096 (112·1229–220·9776)	5·6255 (4·0817–7·9069)
North America	21·0783 (16·1285–30·9608)	66·0197 (50·7840–99·7413)	2·2992 (1·6701–3·3432)
Eastern Europe	7·8326 (6·2571–9·8118)	65·2106 (50·9055–84·6727)	2·2749 (1·8017–2·9094)
Western Europe	15·6651 (11·3721–20·1230)	58·2175 (41·0517–75·8866)	2·0665 (1·4470–2·5908)
Central Europe	8·3560 (6·4831–10·5992)	57·7440 (43·5435–72·6766)	2·0795 (1·5600–2·5592)
Southern Sub‐Saharan Africa	6·3628 (4·9125–7·8282)	33·6285 (25·9907–39·0645)	1·4286 (1·1162–1·6328)
Southern Latin America	5·2630 (3·8651–7·1943)	32·5423 (23·5926–45·1229)	1·2776 (0·9299–1·7071)
Tropical Latin America	5·5221 (4·1212–6·5629)	27·6028 (20·9512–30·5295)	1·0908 (0·8430–1·1969)
Central Asia	3·6565 (3·1654–4·5980)	20·3091 (17·9108–25·5949)	0·7797 (0·6910–0·9922)
Central Latin America	3·2885 (2·4786–4·3273)	17·7484 (13·3281–23·6114)	0·7234 (0·5479–0·9377)
Andean Latin America	2·8237 (2·3764–3·5472)	16·6716 (14·4629–20·9201)	0·7467 (0·6502–0·9372)
Oceania	2·5894 (1·8818–3·8080)	15·8802 (11·3658–24·2364)	0·3597 (0·4788–0·9624)
Caribbean	2·8244 (2·4326–3·6637)	14·6916 (12·7388–18·8543)	0·5985 (0·5202–0·7801)
Central Sub‐Saharan Africa	2·1923 (1·4123–3·2689)	12·6024 (8·4114–19·7562)	0·4690 (0·3047–0·6881)
Eastern Sub‐Saharan Africa	2·1221 (1·6561–2·6536)	11·5941 (9·0618–14·4732)	0·4628 (0·3671–0·5796)
Western Sub‐Saharan Africa	2·2212 (1·7434–3·0505)	10·6301 (8·5621–14·7869)	0·4833 (0·3906–0·6635)
North Africa and Middle East	1·6660 (1·4458–2·2201)	10·4575 (9·2637–14·3409)	0·4237 (0·3753–0·5721)
East Asia	1·4307 (1·0586–1·7655)	10·2859 (7·5003–11·5445)	0·3731 (0·2953–0·4085)
Southeast Asia	1·3100 (1·0986–1·7015)	9·2136 (7·8837–11·9697)	0·3352 (0·2855–0·4343)
Asia Pacific	0·7130 (0·5524–0·9494)	7·7359 (5·7688–10·8982)	0·3089 (0·2295–0·4173)
South Asia	1·1088 (0·8943–1·4254)	6·4210 (5·5115–8·3923)	0·2313 (0·2014–0·2954)

CI, confidence interval.

**Figure 1:**
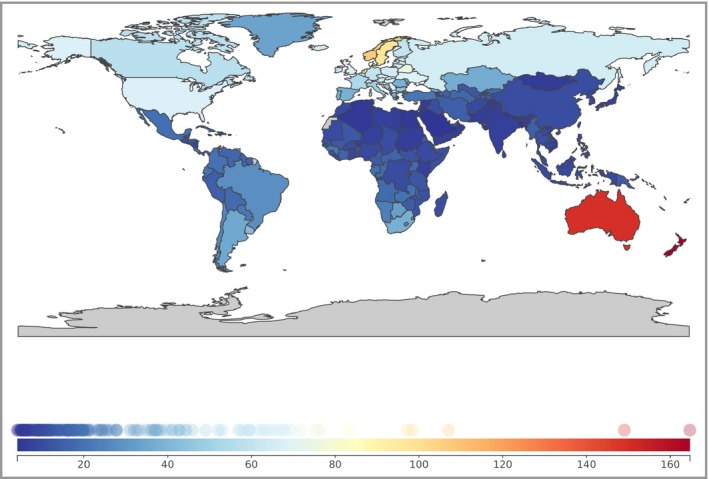
World map of age‐standardized disability‐adjusted life year rates per 100 000 persons from melanoma, both sexes.

Of the 195 countries studied, the five highest age‐standardized incidence rates were in New Zealand [54 (95% CI 39–73)], Australia [54 (95% CI 41–78)], Norway [26 (95% CI 18–32)], Sweden [26 (95% CI 20–35)] and the Netherlands [25 (95% CI 17–30)]. The top‐five highest age‐standardized DALY rates were in New Zealand [165 (95% CI 119–228)], Australia [149 (95% CI 111–221)], Norway [107 (95% CI 70–133)], the Netherlands [98 (95% CI 65–120)] and Sweden [97 (95% CI 71–135)] (Fig. 1). Age‐standardized mortality rates were also highest in New Zealand [6 (95% CI 4–8)], Australia [6 (95% CI 4–8)], Norway [4 (95% CI 3–5)], Sweden [4 (95% CI 3–5)] and the Netherlands [3 (95% CI 2–4)].

**Figure 2:**
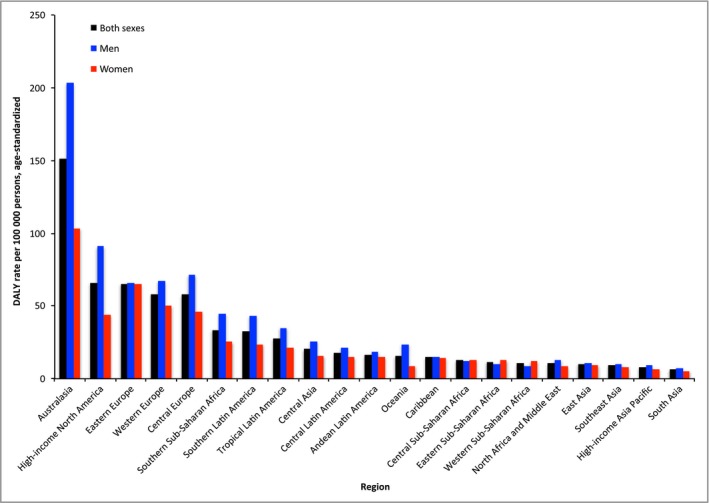
Age‐standardized melanoma disability‐adjusted life year (DALY) rates in 21 world regions by sex.

**Figure 3:**
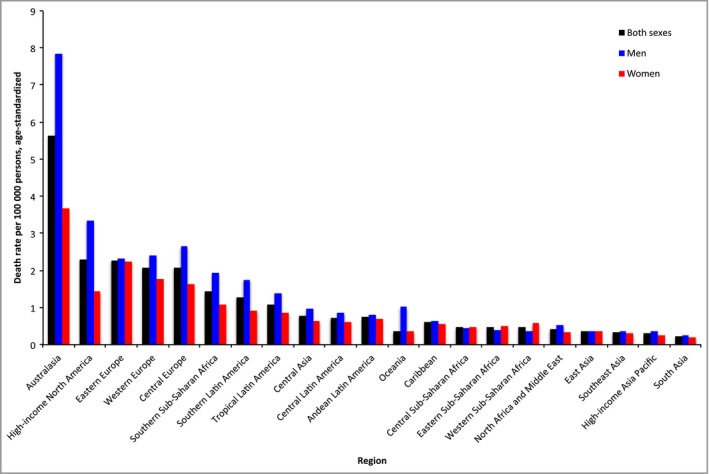
Age‐standardized melanoma mortality rates in 21 world regions by sex.

The global age‐standardized DALY rates resulting from melanoma were 27 (95% CI 18–38) in male patients and 19 (95% CI 17–21) in female patients. DALY rates were greater in men than in women in all world regions with the exception of Central, Eastern and Western sub‐Saharan Africa, where melanoma DALY rates were greater in women (Fig. 2). Differences in melanoma mortality rate by sex followed a similar pattern to DALY rates (Fig. 3). For DALY rate by age, the following groups demonstrated the highest rates: 75–79 years, 70–74 years and ≥ 80 years (Table 3).

**Table 3:** Global melanoma disability‐adjusted life year (DALY) rate per 100 000 persons over human lifespan, ages ranging from 15 to ≥ 80 years, both sexes

Age range (years)	DALYs per 100 000 persons (95% uncertainty interval)
15–19	2·6 (2·3–3·2)
20–24	4·9 (4·2–6·1)
25–29	8·3 (7·1–10·3)
30–34	12·5 (10·6–15·6)
35–39	18·7 (15·9–24·0)
40–44	26·5 (22·1–33·2)
45–49	32·7 (27·2–40·9)
50–54	46·3 (37·5–59·6)
55–59	57·6 (45·9–72·8)
60–64	66·0 (51·2–83·7)
65–69	68·4 (52·4–84·7)
70–74	84·0 (64·1–103·1)
75–79	84·7 (64·2–101·8)
≥ 80	84·0 (64·5–96·5)

## Discussion

We reveal that the greatest burden from melanoma falls on New Zealand, Australia, Europe, the elderly and male populations. Reasons for the disproportionate burden of melanoma in Australasia have been well documented and include a predominantly fair‐skinned population, living with high ambient solar ultraviolet (UV) radiation levels, and having a cultural emphasis on tanning.^16^ A similar study investigating burden of disease metrics for 27 cancer groups in 12 world regions found the highest DALY rates from melanoma in Australia and New Zealand, followed by Northern Europe, North America, Western Europe, Southern Europe and Eastern Europe.^3^ These findings agree well with those of our current study. In addition, the burden of melanoma on many regions of Europe, particularly Western Europe, has been recognized. A prior study in the Netherlands found that from 1991 to 2010 there were 96% and 75% increases in melanoma DALYs in men and women, respectively.^4^ Specific to Scandinavian populations, despite living with low ambient UV, the higher incidence of melanoma may be predominantly attributed to a high‐risk phenotype (fair skin, hair and eye colour), combined with a tanning culture favouring high levels of UV exposure with sunny holidays and indoor tanning.^17^, ^18^, ^19^ Regarding age differences, the higher DALY rates observed in elderly populations are likely a result of peak incidence rates owing to prolonged lifetime cumulative risk factors.^20^ Sex differences in melanoma incidence and mortality are well documented, as men tend to have worse sun protection behaviours and reduced skin screening, as well as biological differences in tumours.^21^

It is of interest to compare the differences in melanoma burden between three high‐income countries, i.e. Australia, New Zealand and the U.S.A., particularly in regard to national policy efforts. Of the 195 countries, New Zealand and Australia ranked first and second, respectively, while the U.S.A. was ranked 18th in melanoma DALY rates. Remarkably, the age‐standardized melanoma DALY rate in New Zealand was almost 2·5 times greater than that in the U.S.A. The burden of melanoma in the Australasian region can at least partly be attributed to predominantly fair‐skinned populations with high ambient UV and cultural habits of outdoor recreation and sun tanning.^22^ However, Australia has undertaken aggressive and comprehensive skin cancer awareness campaigns for over three decades to reduce the burden of skin cancer.^16^, ^23^ Following the International Agency for Research on Cancer classification of UV‐emitting tanning devices as class I ‘carcinogenic to humans’, Australia became the second nation after Brazil to enact a nationwide ban on commercial tanning beds.^24^, ^25^ In contrast, New Zealand has lagged behind Australia in skin cancer prevention efforts and implementation of protective behaviours. Tanning beds are not currently partially or fully banned and a recent study revealed 176 businesses nationwide offering commercial tanning beds to consumers.^26^ In addition, regarding the paediatric and adolescent populations, which spend high‐UV hours at school, multiple investigations found that only 50% of New Zealand schools had an established sun policy behaviour and that sun barriers such as sunscreen and hats were poorly used at outdoor school activities.^27^, ^28^, ^29^ Researchers from these studies note that while multiple nongovernmental charities have been actively involved in sun protection efforts, there is a need for New Zealand government collaboration to promote universal sun awareness and protection policies.^28^

Regarding national melanoma treatment guidelines, a review article comparing the U.S.A., Canada, Europe, Australia and New Zealand, found that the Australian Cancer Network (ACN), responsible for guideline generation in Australia and New Zealand, produced the strictest follow‐up recommendations for patients with melanoma of any stage, including the use of ultrasound in surveillance of lymph node recurrence.^30^ While the National Comprehensive Cancer Network (NCCN) does not issue a consensus on routine melanoma screening, the ACN has recommended against population‐based screening. The ACN and NCCN generally share recommendations regarding biopsy and excisions guidelines.^30^

The GBD relies on the premise that estimates based on high‐quality analyses and prediction models, while imperfect, are better than no data at all. Estimations for areas lacking cancer data are dependent on covariate selection and regional patterns. This highlights the importance of a global effort to improve vital registration systems and cancer registries. There are a number of limitations to the use of the DALY in regard to melanoma. As mentioned above, because the stage of diagnosis is not incorporated, melanoma DALYs may change with time, as increased early detection leads to a higher proportion of detected melanomas at an early stage. Similarly, with recent progress in advanced melanoma treatment and prognosis, the disability from melanoma is likely to change over time, making temporal DALY comparisons difficult.

A recent report investigated DALYs from melanoma by stage among a large cohort of patients from the Belgian Cancer Registry.^31^ Melanoma mortality, expressed as YLLs, was divided among the melanoma stages as follows: 28% stage I, 33% stage II, 26·2% stage III and 13% stage IV. Over half of melanoma morbidity, expressed as YLDs, was attributed to melanomas with node metastases, while 35% was attributed to localized melanomas and 13% to melanoma with distant metastases. For GBD 2015, stage of diagnosis was not incorporated into the GBD disability estimates. Melanoma stage of diagnosis could be included in future GBD 2015 data for high‐income countries. Another area of consideration in future GBD iterations is the particular burden imposed by early detection and preventative activities. Regardless of its shortcomings, the GBD provides high‐quality, comparative estimates of melanoma burden. As GBD results are now produced on an annual basis, the global collaboration continually works to improve existing estimates and incorporate new studies. Epidemiological assessments such as the GBD have the potential to influence research and public policy regarding melanoma pathogenesis, prevention, diagnosis and treatment.
